# The PLOS “Monitoring Universal Health Coverage” Collection: Managing Expectations

**DOI:** 10.1371/journal.pmed.1001732

**Published:** 2014-09-22

**Authors:** The PLOS Medicine Editors

## Abstract

The PLOS Medicine editors introduce the PLOS Collection on Monitoring Universal Health Coverage and discuss the challenges ahead in implementing, monitoring, and evaluating UHC.

*Please see later in the article for the Editors' Summary*

This week, *PLOS Medicine* publishes the PLOS Collection “Monitoring Universal Health Coverage” [1], launched on September 22^nd^ at the Rockefeller Foundation as a side event of the United Nations General Assembly in New York City.

The high profile of the Collection launch is fitting for the topic that has emerged as a frontrunner of the post-2015 agenda and the concept of which has been integral to founding United Nations principles: Universal Health Coverage (UHC) is firmly based on the 1948 WHO constitution that declared health a fundamental human right and also on the Health for All agenda set by the Alma-Ata Declaration in 1978 [2].

The subject of several recent WHO World Reports and World Health Assembly resolutions [3]–[5], over the past few years, UHC has been the focus of much work and effort by the international community in order to turn the broad aims of UHC into an actionable framework. The PLOS Collection adds to the global conversation and consensus by providing the technical details and country-level experience of the implementation and of the monitoring and evaluation (M&E) of UHC.

According to the definition used in the PLOS Collection [6], UHC is the desired outcome of health system performance, whereby all people who need the full spectrum of health services (that is, promotion, prevention, treatment, rehabilitation, and palliation) receive them according to need, without resulting in financial hardship (including possible impoverishment caused by out-of-pocket payments) because of any associated health care costs.

Organized by WHO and the World Bank, and externally peer-reviewed by independent experts, the PLOS Collection explains and discusses these essential and interlinked components of UHC and includes an overview [6], five technical papers [7]–[11], and 13 country case studies (from Bangladesh [12], Brazil [13], Chile [14], China [15], Estonia [16], Ethiopia [17], Ghana [18], India [19], Singapore [20], South Africa [21], Tanzania [22], Thailand [23], and Tunisia [24]) on progress towards the M&E of UHC in each country written by national experts. The PLOS Collection includes a summary of each country case study with the full paper of each provided as supplementary information.

The five technical papers provide insights into current international thinking around the essential UHC components—coverage, financial protection, and equity—and how these might be monitored and evaluated in practice.

Relating to coverage, Ties Boerma and colleagues [7] discuss coverage for the full spectrum of health services as per the definition of UHC and propose that a comprehensive set of core indicators, which can be adapted to each country situation, should be monitored on a regular basis as part of health systems performance assessments. Marie Ng and colleagues [8] explain the concept of effective coverage in which need, use, and quality can be combined into a “data-rich” metric to measure the extent to which an intended health benefit is provided by a specific intervention.

As for financial risk protection, Priyanka Saksena and colleagues [9] examine existing measures that capture the lack of such protection—catastrophic health expenditure and impoverishment—and propose two additional measures. They also recommend regular reliable household expenditure surveys to help monitor any financial risk caused by seeking health care. Knut Lönnroth and colleagues [10] provide the example of tuberculosis to show how a framework for the monitoring of both health coverage and financial protection might work in practice.

Equity is the ethos of UHC, and so, measuring inequality is a main focus of progress towards this crucial component. Ahmad Hosseinpoor and colleagues [11] propose that global monitoring of progress towards UHC should include at least two dimensions of inequality, such as economic status and urban or rural residence, in addition to sex, when appropriate.

The 13 country case studies strikingly illustrate the difficult practicalities of implementing the components of UHC [1]. Although very different and at various stages of the journey towards UHC, the detailed accounts of how each country is grappling to provide its citizens with UHC and monitor progress, while evaluating impact, give in-depth insight into the challenges ahead and the lessons already learned.

Finally, in the Collection overview [6], Ties Boerma and colleagues build on the recently published WHO/World Bank UHC monitoring framework [25] and highlight the key policy messages from the PLOS Collection, such as the need to embed the monitoring and evaluation of UHC into the regular overall health performance reviews that already exist in most countries.

As with other three-word initiatives and their accompanying acronyms that have gone before it, such as Health System Strengthening (HSS) and Primary Health Care (PHC), it is perhaps unfortunate that UHC needs such in-depth explanation to be fully understood by the health community and by policy-makers. There is a real risk that this near-ubiquitous term may be misinterpreted and misunderstood by the very populations and communities that it ought to serve. Consequently, much work remains to be done in a different “M&E” of UHC—managing expectations.

For a start, it might be confusing that the WHO/World Bank target for UHC is not universal, that is, it is not complete coverage: while the target of financial protection from out-of-pocket payments for health services is a laudable 100% coverage to be reached by 2030, the target for essential health services coverage for the same time period is 80% [6],[25]. As with the Millennium Development Goal (MDG) targets, while population proportions may be pragmatic landmarks, providing for the individual remains the ideal. In the drive to achieve manageable targets, while policy makers may be satisfied with such a compromise, citizens may need to understand that their individual health needs may not be met within the target timeframe.

And what constitutes essential health services may also be misconstrued given potential different interpretations of “essential,” especially within the context of the wider spectrum of health services included in UHC [6]. For example, some essential elements such as rehabilitation might get less attention than others, which would be disappointing given the scale of the need for such services and also for the associated financial protection: over 1 billion people currently experience some form of disability, and generally have poorer health and higher rates of poverty than people without disabilities because of the lack of services available to them and the many obstacles they face in their everyday lives [26].

While the momentum towards UHC is set to grow and galvanize in the coming years, coordination with other key global health movements and action plans is also crucial to avoid the much-criticized silo approach of the MDGs. For example, there is currently a global action plan for noncommunicable diseases [27], and also for mental health [28], with accompanying targets and goals. Perhaps UHC could be the umbrella of concerted action and integrated monitoring and evaluation in these areas, rather than a perceived separate entity.

As illustrated in the country case studies in the PLOS Collection [1], despite a tremendous amount of political will, hard work, and effort, UHC is a distant aspiration for many global citizens. While there will be many milestones along the path to fully realizing UHC, it would be disappointing if UHC was considered as simply a utopian construct. Providing equitable access to health services for those who need them while striving to eliminate a major cause of poverty—excessive payments for healthcare—is a crucial catalyst to enable and empower individuals, communities, and populations not only to survive but also to improve their health and quality of life. The next essential step is to ensure that the global population—that is, all potential health care users—comprehend what is meant, and just as importantly, what is not meant, by UHC in their individual situations.

The *PLOS Medicine* editors look forward to the continued conversation around UHC and we hope that you find this PLOS Collection Useful, Helpful, and Clarifying.
